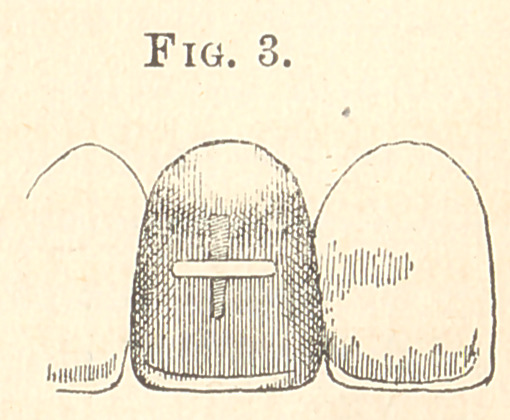# Replacing Porcelain Facings

**Published:** 1892-05

**Authors:** E. B. White

**Affiliations:** Syracuse, N.Y.


### REPLACING PORCELAIN FACINGS.

BY E. B. WHITE, D.D.S., SYRACUSE, N. Y.

I DESIRE to present briefly a novel, simple, rapid, and yet practical system of replacing the porcelain facings of crowns that have become fractured.

FIG. 1.

FIG. 2.

FIG. 3.

Ever since the advent of artificial dentures teeth have been broken in the mastication of "soft bread," and since faced crowns have become so prominent and essential, we have been racking our brains in order to devise some means of repairing the damage done by the destructive attacks of this seemingly harmless edible without removing the crown or bridge from the mouth.

To the man whose soul is in his work there must come ideas quite as numerous as the hours he works, and in locking them up within himself, instead of sowing them broadcast, he is outraging his privilege and duty to the public as well as to the professional fraternity. We are all educators, and human, if not professional, ethics demand that we co-operate for mutual enlightenment and advancement.

Our patients expect us to keep in touch with each other, so that, when necessary, another may intelligently begin where one has stopped.

Our crowns and bridges go to all parts of the world, and knowing that, should an accident occur, they may be properly cared for, because we have exchanged thoughts and experiences with our antipodal brother, should be a sufficient reward for imparting our ideas to others.

In submitting the following method, I feel confident of the approval of those to whom the repair of fractured facings has heretofore been a source of annoyance: After a suitable facing has been selected, drill a hole through the bridge or crown and enlarge it with a small fissure-bur to a horizontal slot (Fig. 1), of sufficient width and length to admit of the pins on facing and in proper position to allow the facing to be fitted to place. After grinding the facing to fit, bend the ends of the pins together and solder, forming a loop (Fig. 2) of sufficient length to reach nearly through or to within about a line of the inner surface of the gold. Then, with a fissure-bur, make a groove on the inner surface of the gold, slightly larger at the upper than at the lower end, at right angles with and across the centre of the slot (Fig. 3), of proper depth to insert a pin through the loop on facing. Make a tapering pin to fit the groove, and after filling the slot and groove and covering the anterior surface of the gold—which now serves as the backing to the porcelain—with creamy cement, place the facing in position and insert the pin in the groove and loop with force enough to make it tight and the facing solid. After the cement solidifies, grind the pin even with the gold and polish.

This makes a most strong and durable repair without any possibility of the porcelain twisting or getting out of place, and can be done in thirty minutes.

## Figures and Tables

**Fig. 1. f1:**
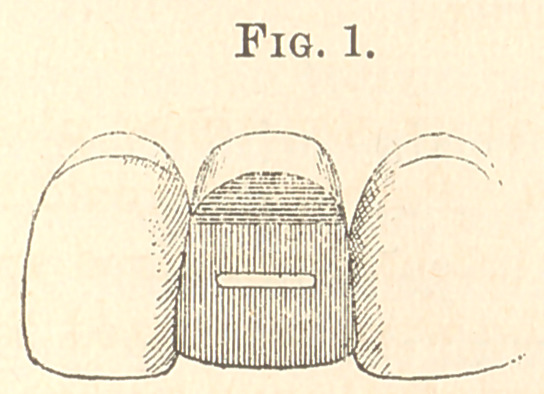


**Fig. 2. f2:**
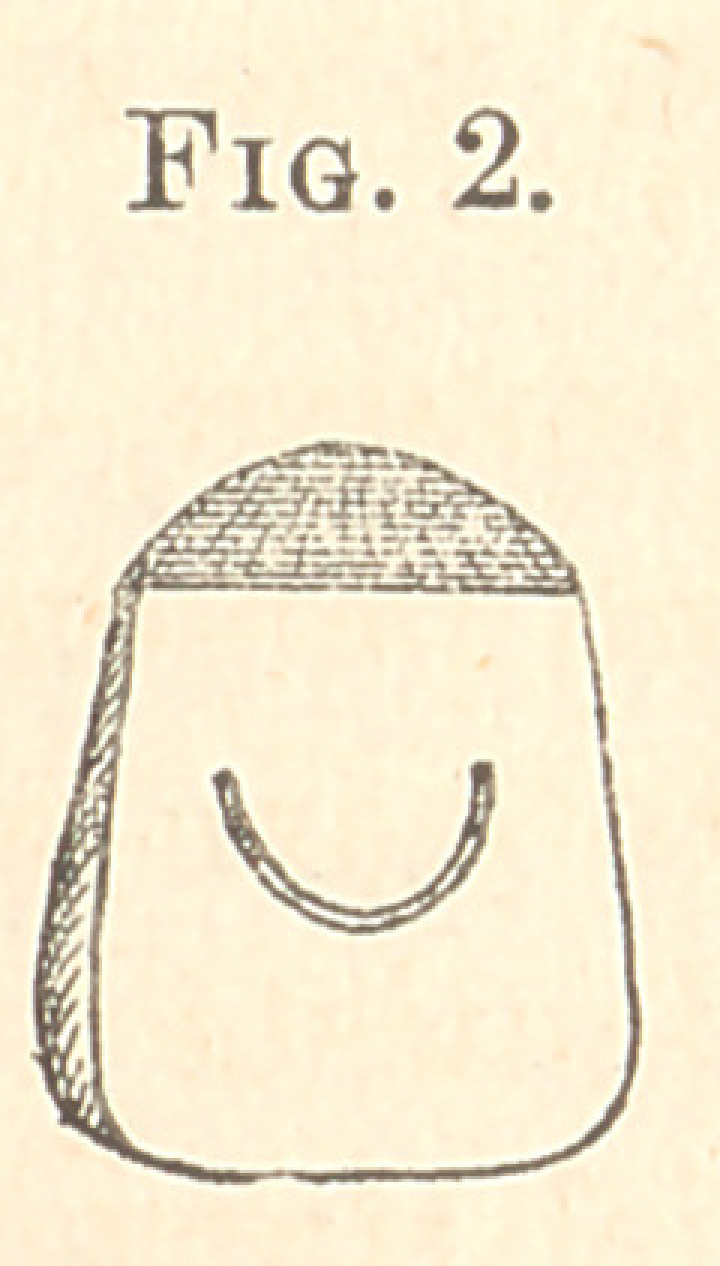


**Fig. 3. f3:**